# Phase-Based Care in Community Mental Health: A Cost-Effective Innovation Using Algorithms, Rating Scales and Treatment Teams for Depression Management

**DOI:** 10.1007/s10597-024-01303-5

**Published:** 2024-06-25

**Authors:** Jules Rosen, Michelle Hoy

**Affiliations:** 1 Summit Behavioral Health Consultants, Breckenridge, USA; 2 Care Connect, LLC, Whitewater, CO USA

**Keywords:** Mental health, Waitlist, Wait time, Delays in care, Behavioral health wait time, Community mental health, Federally qualified health centers

## Abstract

This retrospective, observational report describes an innovative quality improvement process, Phase-based Care (PBC), that eliminated wait times and achieved positive clinical outcomes in a community mental health center’s (CMHC) mood disorder clinic without adding staff. PBC accomplishes this by eliminating the ingrained cultural practice of routinely scheduling stable patients at rote intervals of 1–3 months, regardless of clinical need or medical necessity. Based on four organizational transformations and using mathematical algorithms developed for this process, PBC re-allocates therapy and medical resources away from routinely scheduled appointments and front-loads those resources to patients in an acute phase of illness. To maintain wellness for patients in recovery, lower frequency and intensity approaches are used. This report describes the development of the PBC methodology focusing on the Rapid Recovery Clinic (RRC) comprised of 182 patients with a primary diagnosis of a mood disorder, the largest of the 14 PBC clinics created. Over an 18-month period, wait times were reduced from several months to less than one week and recovery rates, meaning no longer in an acute phase, were 63% and 78% at weeks 6 and 12, respectively for patients who engaged in the program.

## Introduction

Access to behavioral health care in the U.S. is a public health crisis with wait times of 2 months or more, especially for patients on Medicaid (CCBHCs, 2023). Delayed care is associated with symptom deterioration while waiting, increased dropout rates and no-shows once care starts (Biswas, et al., 2018), excessive burden for adult and pediatric primary care providers (Cama et al., 2017; Jetty et al., 2021) and emergency departments (Karaca & Moore, 2020), and greater health care costs (Catarino et al., 2023).

The standard explanation for these backlogs is ‘inadequate staffing’ (Cama et al., 2017; CCBHCs, 2023; Designated Health Professional Shortage Areas Statistics:, 2023; Gates & Mohiuddin, 2022; Zimmerman, 2022). However, the innovation “Phase-based Care” (PBC) demonstrates that a partial solution to this problem is within the reach of community mental health centers (CMHCs) and federally qualified health centers (FQHCS). PBC requires no additional staff, engages patients without delays, and may increase revenue by reducing no-shows. To implement PBC, pervasive, traditional practices of CMHCs must be modified or eliminated.

## Background

PBC was developed through a yearlong Lean Six Sigma consultation engaging with a multi-disciplinary innovation team at a CMHC in western Colorado. The objective was to reduce extensive waitlists and achieve positive clinical outcomes by rapidly providing care of the modality and intensity specific to each patient’s needs. The first task was to identify potentially remediable organizational practices of CMHCs that divert staff resources away from promptly evaluating and engaging with new and acute patients. The team identified the widespread cultural pattern of scheduling appointments at routine 1–3 months intervals regardless of clinical acuity, even after years of sustained stability, as a major contributor to the problem. Examination of our own internal practices revealed ~ 50–70% of the providers’ (psychiatrists and nurse practitioners) and therapists’ clinical hours were usurped by routinely scheduled stable patients, with no-show rates of close to 50% among that cohort.

The innovation team proposed five transformative changes to the traditional mode of scheduling and engaging patients, with the goal of shifting ~ 70% of provider and therapist resources to those who are new and/or acute. The following modifications were initiated: (1) weekly clinics, (2) multi-disciplinary teams, (3) measurement-based care and data monitoring, (4) adjusting care plans as acuity changes, (5) using algorithms to adjust staffing levels.

### Weekly clinics

Patients agreeing to participate in PBC are assigned to weekly clinics that shift 70% of the providers’ and therapists’ time to new and acutely ill patients. Scheduled appointments for therapy and/or med management as well as the option for same-day walk-ins (SDWI) assure access to a team member when the patients feel engagement is needed. During the one-hour weekly clinic staff meeting, without patients present, the teams review the clinical progress and resource utilization of the acute phase patients from the previous week, plan for the new scheduled admissions for the current week, and review the anticipated SDWI patients.

### Multi-Disciplinary Teams

CMHCs employ staff across multiple disciplines, however, collaborative care within CMHCs is fraught with cultural obstacles and not commonly practiced (Durand & Fleury, 2021). In most PBC clinics, a case manager serves as the “air traffic” controller, managing phone calls, triaging SDWIs, and coordinating the workflow for all staff during the clinic. During the weekly review of acute patients, team members question each other regarding the effectiveness of various treatment modalities. For example, if a patient is regularly scheduled for psychotherapy without clear therapeutic goals and indication of clinical progress, the team will query the therapist if the patient would have her needs met by working with a case manager and or attending a life-skills group.

### Measurements and Data

Integrating quantitative data into clinical care at CMHCs is a cultural transformation. In PBC clinics, self-administered rating scales are obtained at every touch and incorporated into all therapeutic interactions. These quantitative measurements, along with clinical information, guide the treatment teams in their discussions of acuity phase and treatment plan modifications.

### Weekly Team Meetings Modify Care Plans in “Real Time”

Typically, in CMHCs, care plans are reviewed and adjusted according to rote time intervals dictated by state regulations; not by patient progress, or lack thereof. Understanding that most psychiatric illnesses have “phases” ranging from high acuity to full remission (Cosci & Fava, 2013; Kupfer, 1991), the acuity levels and resource utilizations of all new and acutely ill patients are reviewed during the one-hour PBC weekly team meetings. The designated phases are “acute”, “recovery”, and “maintenance”. The acute phase typically lasts 4–12 weeks, depending on diagnosis, symptom severity, and response to treatment. A recovery phase of 6 months following the acute phase offers fewer therapy and provider resources, unless symptoms re-emerge. The maintenance phase provides continuity of care for stable patients after recovery for those with chronic or recurring illnesses and allows immediate access in case of relapse. Time-limited, goal-focused psychotherapy is available to all patients, regardless of phase.

### Algorithms to Guide Staffing

Algorithms developed for PBC are used to inform the level of staffing of each discipline required to serve that clinics’ patient population. Key input data for these algorithms include admission rates (new referrals per week) and projected treatment needs from each discipline during all phases of illness. For example, a PBC with high numbers of people with schizophrenia will be projected to need more case management than psychotherapy hours. When initially designing a PBC clinic, staff provide their “best guess” regarding the *average* number of anticipated new or acute patients per week based on historical data, the anticipated *average* number of sessions required to achieve recovery, the *average* acute phase duration, and *average* resources needed to maintain wellness for patients in recovery. Those initial numbers entered into the algorithm demonstrates to an organization, often skeptical, how a PBC clinic can accommodate a defined population *without* additional staff. As PBC clinics incrementally grow with new patients weekly, the algorithms are adjusted according to the actual data of new referrals, resource utilization, and rates of recovery over at 3–4-month intervals.

## Methods

When planning the Rapid Recovery Clinic (RRC) for mood disordered patients, the team initially projected 4 new admits per week; 60% needing psychotherapy and 80% requiring medication management. The average hours for therapy and med management to reach recovery was projected to be 5 and 2, respectively. Case management use was projected to be 20% with an average of 1.5 h. Based on those proposed figures, the algorithm indicated that the RRC would need 10 therapist hours, 6 provider hours, and 1 case management hour per week. The actual data, generated after 9 months of clinic operation, differed and the algorithms were revised. There were only 3 admissions per week, only 40% needed or wanted therapy, 85% needed medication management and 30% required case management. Hours to recovery were 3 for therapy, 2 for medication management and 3 for case management. Based on the actual clinical data the algorithms indicated only 4 therapy hours (not 10), 5 provider hours (not 6), and 3 case management hours (not 1) were needed each week.

This report describes the development and an 18-month outcomes evaluation of the Rapid Recovery Clinic (RRC) for mood disorders in Grand Junction, CO. As required by the Quality Assurance Committee review, the data used for this analysis were de-identified, collected solely for the clinical care of the patients and process improvement for the clinic, and without control group or randomization. IRB approval was not required.

### Rapid Recovery Clinic (RRC)

Following an intake evaluation, patients with non-psychotic, non-bipolar mood disorders and PHQ 9 scores ≥ 14 were invited to engage in the RRC or pursue treatment as usual, which typically resulted in an 8–12 week delay.

Over an 18-month period, from May 2017–November 2018, 284 patients met inclusion criteria and agreed to enroll in the RRC. Approximately 36% of enrolled patients attended only one clinic (n = 102) and are not included in this data analysis, as by definition, they were not engaged in treatment. Of the 182 patients who attended at least two clinics, 13.2% (n = 24) dropped out prior to week 8, without information regarding their clinical outcome and are not included in the outcomes data analysis. This report includes the 158 patients who *engaged* in the RRC for at least two visits.

In terms of patient characteristics, ~ 90% had a primary diagnosis of major depressive disorder, and more than 70% had co-diagnoses including substance abuse, PTSD or GAD. Females made up 60.7% of the patients, 37.3% graduated high school and 53.1% had education beyond high school. Mirroring the racial and ethnic characteristics the community in Grand Junction, Colorado, 89.2% were white, 8.2% Hispanic and 1.8% other. No African American participants enrolled.

Within one week of completing their intake evaluations, patients participating in the RRC could meet with their treatment teams during the weekly clinic to develop a single, multi-disciplinary care plan. As a mood disorder clinic, the Patient Health Questionnaire-9 (PHQ-9) was completed and discussed with the patients at each encounter (Kroenke et al., 2001). While the PHQ9 assesses symptom frequency over the prior two weeks, modification was allowed to explore changes over a single week to spark clinical exploration and discussion. The consensus opinion of the treatment team was that an average PHQ-9 score ≤ 10 corresponds to likely resolution of an acute phase and was the “objective” measure of “recovery” for this report. Of note, “recovery” is not the same as remission, and additional treatment, albeit lower intensity, may still be needed.

The duration of the acute phase of 12 weeks, as described by Kupfer (1991), was chosen by the team to evaluate recovery rates for this report. Therapeutic touches, defined as attending a weekly clinic and engaging clinically with any staff member, were reviewed at the treatment team meetings for acute phase patients.

A paired t-test was used to compare pre- and post-PHQ 9 scores. The “post” score was defined at the first visit that the PHQ-9 was ≤ 10, or by the score obtained during the visit closest to week 12. Patients who achieved recovery early but relapsed before week 12 were not considered to be recovered.

Guided by the algorithms using actual data and the clinical wisdom obtained from the first 8 months following clinic inception, the clinic staffing levels during this 18-month period typically included a psychiatric nurse practitioner (4 h), psychiatrist (2 h), therapist (4 h adjusted to 6 as needed), medical case manager (4 h), case manager (4 h) and peer support specialist (4 h). This is similar, but not identical, to the “actual” algorithm generated numbers, as various staff had additional responsibilities within the RRC not reflected by the predicted patient contact hours, such as facilitating education and therapy groups during clinic hours.

## Results

The mean pre- and post-PHQ-9 scores were 20.3 (SD 5.2) and 12.0 (SD 7.2), respectively for the 158 engaged patients (t = 13.1, p < 0.0001, df = 129). At week 6, 63% of engaged patients achieved recovery, and 78% by week 12. The mean time to first appointment with both psychiatry and therapy was 3–5 days, and the drop-out rate for the engaged patients prior to week 12 without recovery was 13%.

An average of 2.3 therapy hours (1–8), 1.4 medication management hours (1–2.5), and 0.5 h for case management were utilized to achieve recovery or by week 12, with an average of 4.3 total “touches” (not hours), including peer specialists and group engagement. For the 18 months during which this data was collected, the RRC typically served 132 active patients in all phases of acuity, with 36 in the acute phase at any given time.

Of the patients who engaged in the acute phase, 134 (84.8%) continued with the RRC during the recovery phase, with at least one visit over the subsequent 6 months. Relapse within 6 months was reported in 14 (10.4%) with two patients requiring hospitalizations.

Of the total of 284 patients admitted in the RRC, 44.4% either dropped out or disengaged prior to recovery or by week 8. With a recovery rate of 78%, PBC treatment for depression is highly effective for *engaged* patients; however, the rate of recovery for all enrolled patients, or intent to treat, is 43.4%.

## Discussion

Shortages in the behavioral health workforce clearly contribute to delays in care, especially for clinics serving under-insured and Medicaid consumers. However, as demonstrated by the RRC, PBC can eliminate waitlists and wait times, indicating that CMHCs can potentially reduce some obstacles to timely access and engagement. CMHCs routinely schedule patients who are stable, many without clear indication of medical or clinical needs. Some states regulatorily require that a psychiatrist or therapist “must see the patient” at intervals not to exceed 90 days (Indiana Health Coverage Programs, 2022). Both culturally and regulatorily driven overuse of behavioral health resources without clinical necessity needlessly add to delays.

The profound cultural changes needed to initiate a PBC clinic requires 4–6 months of weekly consultation with both management and staff. With PBC, a multidisciplinary clinic offers medication management, therapy, case management, groups and peer support in “one stop shopping”, decreasing time and travel burdens for patients (Kim & Jeon, 2019), and offering the opportunity for therapeutic benefits of engaging with staff other than their assigned provider or therapist (Dafsari et al., 2023; Posternak & Zimmerman, 2007). With a single multi-disciplinary care plan, patients wishing to engage in therapy and medications simultaneously, rather than sequentially, may experience more rapid response rates (Dunlop et al., 2019; Khan et al., 2012). The option of SDWI in addition to scheduled services is both effective and desirable to patients, allowing them the opportunity to engage according to their needs rather than waiting for a scheduled appointment (Sarmiento & Reid, 2023).

The introduction of rating instruments into clinical care is novel for most CMHCs. Despite evidence supporting their clinical benefits (Guo et al., 2015; Lewis et al., 2019), only 18% of psychiatrists and 11% of psychologist incorporate rating instruments into their practices (Demyttenaere & Jaspers, 2020; Wood & Gupta, 2017). With PBC, the rating scales serve two purposes. First, symptom elucidated by the ratings are explored in each therapeutic interaction. Subtle changes in the score of a specific symptom may reflect early improvement that might not be perceived by the patient. Secondly, the scores reviewed during the weekly team meetings contribute to the acuity assessment for a patient’s phase delineation, and matching resource allocation with changing clinical needs.

Using a PHQ 9 score of ≤ 10 was chosen by the treatment team as a marker of “recovery”. While a PHQ 9 < 10 is typically used to indicate a patient is unlikely to meet criteria for MDD in research programs (Kroenke et al., 2001), there is substantial heterogeneity depending on setting and diagnostic assignation, and several studies have identified scores above 10 to have greater specificity and sensitivity in distinguishing syndromal and subsyndromal depressions (Gilbody et al., 2007).

The algorithms are essential to project clinic staffing needs, and to monitor, in real time, if staff are working according to their optimal licenses. The disparity in algorithm’s projections during the planning phase and the “actual” staffing based on data is predominantly driven by the psychotherapy and case management domains. Only 40% of the patients, not 60% as proposed, opted to engage in therapy, and the actual average number of therapy hours to recovery was 2–3, not 5 as predicted. Case manager hours needed for the clinic increased from 1.5 (proposed) to 3 (actual). These discrepancies, observed in the development of virtually all PBC clinics, are likely due to therapists providing case management and supportive care for many patients who are not actually engaging in psychotherapy.

PBC reduces the negative impact of no-shows. Factors driving high no-shows include long wait times, rigid scheduling policies, and scheduling patients with minimal symptom severity (Catarino et al., 2023; Marbouh et al., 2020; Molfenter, 2013). PBC addresses all those issues. SDWI patients generally offset losses when scheduled patients fail to show. The RRC did not offer telemedicine for scheduled and SDWI appointments, however all post-pandemic PBC clinics have incorporated that option.

Finally, it is important to make clear that the evaluation of the RRC outcomes was not a designed research study with set methodologies and hypotheses. Rather, this was a quality improvement process under the guidance of a Lean Six Sigma (LSS) consultant. Integral to LSS is use of metrics and data analysis as well as flexibility and readiness to change based on real-time assessments of successes and failures (Olutade et al., 2023). There was no control group, no standardized treatment protocols, and no use of structured diagnostic instruments. The RRC data reflects the outcomes of mood-disordered patients working with a clinical team in a community setting, employing an innovative approach that synchronizes resource utilization and the clinical needs of the patients in real-time.

There are other innovations that incorporate features of PBC. For example, Collaborative Care utilizes measurements and a behavioral health team within primary care (Reist et al., 2022), and SDWC offers an alternative to rigid scheduling (Sarmiento & Reid, 2023). We are unaware of other programs that have included all the organizational changes of PBC.

## Limitations

One of the ongoing challenges for PBC is to understand the reasons many patients failed to engage. The “intent to treat” (ITT) rate was below 50%, highlighting that the current PBC model does not resonate with everyone. For the current report, non-ITT analysis is the correct approach as the “aim is to estimate the effect of treatment as delivered or as received”, as opposed to “assigned” under the ITT approach (Ten Have et al., 2008).

This study did not conduct a financial analysis of the RRC compared to traditional CMHC care. However, during clinic hours, therapists’, providers’, and case managers’ schedules were typically fully occupied with either scheduled patients or SDWIs. A subsequent study, now under review, reflects a financial gain of > 30% in Medicaid reimbursement for the 702 wait-listed patients who engaged in clinical care through PBC. Satisfaction surveys were not collected from staff or patients during the specific time the RRC data was collected. These will be included in future programs, with comparisons to traditional treatment modalities.

In conclusion, the current PBC data shows that culture changes that support the reassignment of provider and therapist resources away from routine follow-up care to prioritize the needs of new and acute patients reduces delays in treatment and enhances outcomes. Additional studies are in progress which include control groups, financial analyses based on Medicaid claims, staff and patient satisfaction surveys, and exploration of reasons for failure to engage.
